# Translation and Cross-Cultural Adaptation of Quality of Life Scale in Patients with Onychomycosis

**DOI:** 10.3390/ijerph18115793

**Published:** 2021-05-28

**Authors:** Vasco Silva-Neves, Ana Caramelo, Paulo Alves, Carla Pais-Vieira, Alexandra Palmer Minton, Ana María Rodríguez-Leboeuf, Miguel Pais-Vieira

**Affiliations:** 1 Centro de Investigação Interdisciplinar em Saúde, Instituto Ciências da Saúde, Universidade Católica Portuguesa, Rua de Diogo Botelho, 1327, 4169-005 Porto, Portugal; vneves@porto.ucp.pt (V.S.-N.); caramelo.ana@gmail.com (A.C.); pjalves@porto.ucp.pt (P.A.); cvieira@porto.ucp.pt (C.P.-V.); 2 Hospital das Forças Armadas-Polo Porto, Avenida da Boavista, 4050-113 Porto, Portugal; 3 Patient Centered Solutions, IQVIA, San Francisco, CA 94105, USA; Alexandra.Palmer@iqvia.com; 4 Patient-Centered Endpoints, Patient-Centered Solutions, IQVIA, 28007 Madrid, Spain; AnaMaria.Rodriguez@iqvia.com; 5 Department of Medical Sciences, iBiMED—Institute of Biomedicine, Campus Universitário de Santiago, University of Aveiro, Agra do Crasto—Edifício 30, 3810-193 Aveiro, Portugal

**Keywords:** quality of life, onychomycosis, translation, cross-cultural adaptation

## Abstract

(1) Background: Onychomycosis (OM) is a fungal nail infection, considered a risk factor for diabetic foot ulcers. It is associated with changes in quality of life, in terms of pain, self-confidence and self-esteem. The aim was to translate and adapt the OM quality of life questionnaire “OnyCOE-t^TM^–Quality Of Life Questionnaire Onychomycosis (Nail Fungal Condition)”. (2) Methods: This study followed the guidelines proposed by Beaton et al. (2000), where two English to Portuguese translations were performed and, after an expert consensus, a common version was obtained. This was followed by two back-translations. The expert committee achieved semantic equivalence, idioms and concepts. The pre-test was applied to 49 people. The final version and processed data were sent to the authors. (3) Results: We adapted terms semantically, modified statements syntactically, altering items from interrogative to affirmative. (4) Conclusions: The translated version of the “Quality of life–Onychomycosis” questionnaire suggested that it can be used for further studies to test validity and reliability in this population.

## 1. Introduction

Onychomycosis (OM) is a fungal nail infection, also known as *Tinea unguium* [1], with estimated prevalence of 10–14% in the general population [2,3,4]. Although OM is considered an aesthetic problem [5], it is recognized as a risk factor, promoter of foot ulcers and enhancer of acute bacterial cellulitis, particularly in diabetics [6,7,8]. Despite the difficulty of drugs to penetrate the nail plate, topical treatment is often preferred over a systemic approach [9]. These treatments have contraindications associated with comorbidities, as well as iatrogenic effects [10,11], and a high number of resistance and reinfection cases [10].

Altogether, OM frequently becomes a chronic health problem with a significant impact on the quality of life (QOL) of these people [10]. In addition to OM being responsible for half of all nail disorders [12], it is also considered a skin disorder, with negative social, psychological and occupational effects [13].

According to a recent review, QOL has been evaluated in people with OM, namely in randomized clinical trials [11]. The most commonly used instruments for evaluation of QOL in OM are the OnyCOE-t^TM^, NailQoL and Drake’s QoL Questionnaire [11]. However, the OnyCOE-t^TM^ [14] questionnaire is the only psychometrically validated instrument used to assess response to treatment through patient-reported outcomes (PRO) [15]. This instrument is composed of 33 items, with 6 multi-item scales and 1 single-item scale, and was validated through the IRON-CLAD^®^ (a large, randomized multicenter American clinical trial), present internal consistency reliability by Cronbach’s alpha > 0.84. The Responsiveness was good, regarding the treatment satisfaction, symptom frequency, overall problem and appearance problem (Guyatt’s statistic of 1.72, 1.31, 1.13, and 1.11, respectively) [14,15].

The first study of OM’s impact on patients’ QOL was published in 1993. This was developed to measure the relationship between QOL and specific aspects of OM, specifically, physical (pain), psychological (shame) and social problems (isolation). This study recognized that QOL is negatively affected by OM, namely in terms of mental health, social interaction, self-confidence, self-esteem, physical discomfort and pain. In this study, personal concern and social embarrassment were also found to be present, due to fear of nail exposure [16]. Some studies demonstrate that, although some people with OM can adapt to this condition, a large portion of them continue to report embarrassment, discomfort and significant reduction in QOL [11]. While OM is not a significant impediment to work, some participants have reported losing days of work, with consequent implications [16,17,18].

In Portugal, prevalence studies estimate rates between 17 and 47% [5,19], which is above the 10–14% estimated in the general population [2,3,4]. This discrepancy reinforces the need to study OM in the Portuguese population to understand the health impact, and because a previous study reported that the Portuguese geriatric population considered OM as an aesthetic condition, unless foot problems were present [5].

With this study, it is intended to translate and culturally adapt a questionnaire specifically designed to measure the consequences of OM reported by patients in the “Quality Of Life Questionnaire Onychomycosis–Nail Fungal Condition (OnyCOE-t^TM^)” for the Portuguese population [14]. 

## 2. Materials and Methods

OnyCOE-t^TM^ was translated and adapted according to the guidelines of Beaton et al. [20] (see details in Table 1). The authorization to translate the scale into Portuguese was obtained from the authors of the instrument. The questionnaire was developed to assess the QOL reported by patients with OM. It allows an assessment of symptoms related to nail involvement, using a Likert scale with six items and five response options, which, due to the frequency described by patients, reveal the degree of perceived discomfort caused by this problem. In addition, this questionnaire evaluates problems related to appearance, using eight items with four answer options. It also has a scale problem related to physical activities through seven items and four response options. General problems with one item and four response options are also assessed. The questionnaire also includes a stigma perception scale with seven items and five answer options and, finally, a satisfaction scale with three questions and five answer options [14].

A sample of 49 patients was selected from a larger sample of 90 patients that were involved in a separate OM prevalence study, including all participants that accepted answering the OnyCOE-t^TM^ questionnaire and consented to being interviewed. 

The methodology recommended by Beaton et al. [20] proposed a sequential script of steps to concretize the translation and cross-cultural adaptation. The methodology used was separated into stages (I–VI) which is detailed in Table 1. 

The expert committee (as recommended for stage IV) was selected from the university and hospital staff and composed of an English teacher, three PhD level researchers and/or professors in health sciences, one PhD expert on QOL scales, one PhD student expert in medical-surgical nursing, two American collaborators responsible for the company that owns the original scale.

This study was submitted and approved by the Ethics Committee of Armed Forces Hospital (see Institutional Review Board Statement on page 10).

### Statistical Analysis

Statistical analysis was performed using the IBM SPSS Statistics program (version 26). The sample was characterized, including mean, standard deviation and percentages through descriptive analysis. Lastly, the sample studied was small and did not allow an appropriate analysis of the psychometric characteristics. Therefore, such analysis will be performed in a different study.

## 3. Results

This study was carried out with 49 patients of the Hospital das Forças Armadas, Polo Porto, between January and December 2019. The majority were married 38/49 (78%), lived in the district of Porto (Portugal) 38/49 (78%) and attended podiatry consultations 30/49 (61%). About one-fifth of the sample was diabetic 10/49 (20%). Table 2 shows the demographic and clinical data obtained.

The results of this translation and cross-cultural adaptation process are presented in stages. We highlight only the results adopted after the discussion between translators, researchers and health professionals, in the different stages in which they were involved.

### 3.1. Results of Stages I and II

The initial translations and synthesis were performed in a single version (T12), and the results are summarized in Table 3.

### 3.2. Results of Stage III

In stage III, two back-translations were performed by two translators whose mother tongue was that of the original questionnaire (American English). These translators were not specialists in clinical terminology and did not know the original questionnaire, avoiding translation bias. It was found that the back-translations were direct, with no significant doubts of the terminology. Whenever possible, it was decided to introduce the simplest terms, in order to facilitate the application of the questionnaire to people with less education. Although the two versions that resulted from the back-translation were similar to the original version, some words and/or expressions presented several possible translation options, which are highlighted in Table 4.

### 3.3. Results of Stage IV

In stage IV, the analysis and validation by the expert committee was carried out. After revision and corrections suggested by the owners of the original questionnaire, specialists (teachers/English translators, health professionals and researchers) were consulted. The documents under analysis were sent to all experts, namely the original questionnaire, the T12 questionnaire and the file to assess the identified discrepancies. Suggestions and corrections were requested from each specialist individually, after each opinion the necessary changes were made, and the prefinal version was made available to all. Consensus was reached on all items listed in Table 5.

### 3.4. Results of Stage V

The pretest version was applied to 49 participants. They were asked about the meaning of each item and if they understood what was being asked. The distribution of responses was examined, and items with no response were identified. Overall, 44/49 (90%) participants did not report any difficulty in filling it out. Approximately 5/49 (10%) older participants, with low education, said that the questionnaire was long, and only two questions raised doubts, identified in Table 6.

## 4. Discussion

In this study, a translation and cross-cultural adaptation of the OnyCOE-t^TM^ translated scale was performed. Overall, no major difficulties were found by the researchers and translators in the six-step translation and adaption process. A small number of semantic and syntactic problems had to be resolved. We highlight these items: “Being embarrassed by the appearance of your nails” and “Feeling self-conscious about the appearance of your nails” that generated more doubts since the initial translations were not consensual. This occurred because “Being embarrassed” could be translated as “Estar constrangido/Estar envergonhado”, and “Feeling self-conscious” could be translated as “Sentir-se constrangido/Reconhecer ou tomar consciência”. The versions that, from a semantic point of view, were considered to be easier to understand by Portuguese people (EU) were “Sentir vergonha com o aspeto das suas unhas” and “Reconhecer ou tomar consciência do aspeto das suas unhas”, respectively. From the syntactic point of view, the wording of question two and all associated items were changed from interrogative to affirmative, to facilitate their understanding. We kept question four, referring to the degree of satisfaction (which was optional in the answer), because it may allow for assessment of another dimension in the care of these patients in future studies. 

The final version was named Questionário sobre a Qualidade de vida–Onicomicose (doença fúngica das unhas). This is the first QOL instrument for patients with OM, translated and adapted to Portuguese (EU). In the semantic adaptation, we sought to gain knowledge into the characteristics of the Portuguese population, in particular, regarding the lower levels of literacy, from the perspective of OM’s effect on patients’ QOL. The values that reflect aspects associated with the impact of OM on QOL, shame/embarrassment (53%) and concern (80%), are in line with previous studies [15].

A small, non-representative sample was studied. Therefore, the psychometric characteristics of the translated scale could not be properly evaluated. Performing additional studies, with larger samples and different realities compared to this study, can improve our detailed knowledge on construct validity, reliability and response patterns. In addition, it should be noted that the best cross-cultural adaptation method is still a matter of debate [21]. This means that the results of the present study may be influenced by the particular method chosen.

The present study of translation and adaptation is only the first step to test the psychometric properties of this instrument in larger samples of the Portuguese population. The present questionnaire will allow a better understanding of the impact of OM on patients’ QOL, which may facilitate the identification of more effective and personalized care strategies [22,23]. 

## 5. Conclusions

The translation and cross-cultural adaptation of the OnyCOE-t^TM^ questionnaire, in Portuguese “Questionário sobre a Qualidade de vida–Onicomicose (doença fúngica das unhas)”, suggests that it may be used for further studies to test validity and reliability in this population. The translated questionnaire demonstrated semantic equivalence to the original. The original authors, collaborating experts and the patients with OM demonstrated acceptance of the questionnaire. Further studies with larger samples are required for a full validation of the questionnaire.

## Figures and Tables

**Table 1:** Description of the translation process adapted from Beaton et al. [20].

Stage	Action	Descriptive
I	Initial translation (Translation 1 + Translation 2)	In this stage, two independent translations were conducted, from the original language to the target language, in this case, from American English (USA) to European Portuguese (EU), by bilingual translators, whose mother tongue is Portuguese (EU). One of the translators was considered naïve (not having knowledge of the subject under study), thus allowing a translation that reflects the language used by common sense. In this version, it was expected that different meanings would be detected when compared to the other version. The other translator was familiar with the concepts and meanings of the subject under study (OM). This translation sought to provide more reliable equivalences to clinical traits. At the end of this process, reports of each translation were developed, identifying the ambiguity.
II	Summary of translated versions (T1 + T2)	Here, the translators of the T1 and T2 versions of the OnyCOE-t^TM^ questionnaire met to resolve discrepancies and measure the semantic content, to produce the translated version, T-12, of the translation questionnaire (T1 + T2). The divergences were compared and identified until a consensus was reached, always considering the original questionnaire. This meeting was attended by the researcher responsible for the translation and adaptation of the scale for the Portuguese population, which synthesized the opinions resulting from the two translations, giving rise to the consensual version of the questionnaire. The methodology used at the meeting included a full reading of the original questionnaire, sentence by sentence, in which the translators compared their translated versions, in search of a semantic and syntactic consensus. The responsible researcher moderated the dynamics of the meeting and created the T-12 version. Notwithstanding unanimity in translation, there were some situations in which more than one form of translation was assumed to be correct, which also allowed for changes. This process resulted in the merger of the two versions known as (T-12). As the previous phase, a report was prepared that identified the situations that generated doubts and the options decided.
III	Back-translation	From the T-12 version, two back-translations were performed. The BT1 and BT2 were produced by translators with the mother tongue of the original questionnaire (USA). As in the previous steps, a report was produced for each translated version. This third step allowed confirmation of the validity and ensured that the translated version reflected the content of the original version. It also allowed for detecting the presence of inconsistencies and/or conceptual errors in the primary translation.
IV	Expert Committee Review	The expert committee intended to achieve intercultural equivalence. It was intended to consolidate all versions of the questionnaire and to develop the pretest version. The expert committee consisted of health professionals, translators and language professionals, as recommended by the guideline. They reviewed all translations (T1, T2, T12, BT1, BT2) to reach consensus and eliminated any discrepancies that may have still existed. It was decided to obtain equivalence between the original version and the final version with regard to semantics, languages, life experiences and concepts.
V	Pretest of the Prefinal version	The pretest was applied to a convenience sample of hospital patients with OM (*n* = 49). The requirement of the guideline translation protocol (to be applied to 30–40 people) was met.
VI	Submission of the final version to authors	Although the owners of the instrument have always followed the translation and validation process, in order to comply with the adopted guide, the final version was sent to the authors, and the data were treated in SPSS v.26. This verification of the final version by the owners thus served as an additional measure to ensure that the properties of the original instrument were present in the translation performed.

**Table 2:** Demographic and clinical data.

Age	*n* = 49 (%)
Mean 66 years (SD = 16.24; min 19–max 90)	
≥ 18 ≤ 40	6/49 (12%)
> 40 ≤ 60	13/49 (27%)
>60	30/49 (61%)
Sex	*n* = 49 (%)
Female	16/49 (33%)
Male	33/49 (67%)
Education	*n* = 49 (%)
Basic education first cycle	15/49 (31%)
Basic education second and third cycle	10/49 (20%)
Secondary education	18/49 (37%)
Higher education	6/49 (12%)
Signs and symptoms of onychomycosis	*n* = 49 (%)
Pain	21/49 (43%)
Soreness	33/49 (67%)
Thickening	44/49 (90%)
Yellowing/discoloration	49/49 (100%)
Impact on quality of life	*n* = 49 (%)
Caregiver needed	29/49 (59%)
Daily hygiene for feet	38/49 (78%)
Self-care difficulties	34/49 (69%)
Shame/embarrassment	26/49 (53%)
Concern	39/49 (80%)
Onychomycosis prevalence	*n* = 49 (%)
Onychomycosis for more than 5 years	24/49 (49%)
Laboratory diagnosis (KOH + culture)	32/49 (65%)

**Table 3:** The original and translated proposed version of the OnyCOE-t^TM^.

	Original Version (English USA)	Translated Version (Portuguese EU)
Question 1 (b)	“Soreness, redness or swelling of your toes or toenails”	“Desconforto vermelhidão ou inchaço dos dedos ou das unhas dos pés”
Question 1 (c)	“Thickening or swelling of your toes or toenails”	Espessamento ou inchaço dos dedos ou das unhas dos pés”
Question 2	“During the PAST 4 WEEKS, how much of a problem were the following because of your nail fungal condition?”	“Durante as ÚLTIMAS 4 SEMANAS, em que medida as situações a seguir apresentadas foram um problema e ou dificuldade devido à sua doença fúngica das unhas?”
Likert scale	“Somewhat of a problem?”	“Moderadamente problemático”
Item (c)	“Being embarrassed by the appearance of your nails?”	“Estar envergonhado/a com o aspeto das suas unhas?”
item (d)	“Feeling self-conscious about the appearance of your nails?”	“Sentir-se constrangido/a com o aspeto das suas unhas?”
item (f)	Feeling that people may see you as unclean or untidy?”	“Sentir que as pessoas o/a pudessem ver como pouco limpo/a ou desleixado/a?”
item (i)	“Doing activities that require you to go barefoot in public (such as swimming, going to the beach, getting into a hot tub, or working out at a health club)?”	“Realizar atividades que implicam andar descalço/a em público (tais como nadar, ir à praia, entrar numa banheira de hidromassagem, ou treinar num ginásio)?”
item (j)	“Doing any hobbies that require a lot of time on your feet (such as jogging, golfing, playing tennis or dancing)?”	“Realizar atividades de lazer que implicam estar muito tempo de pé (tais como correr, jogar golfe, jogar ténis ou dançar)?”
item (k)	“Performing daily activities that require you to be on your feet a lot (such as waiting tables, working as a cashier or salesperson, making deliveries, or construction work)?”	“Desempenhar atividades diárias que exijam que esteja muito tempo de pé (tais como servir à mesa, trabalhar como caixa ou vendedor, fazer entregas, ou trabalhar na construção)?”
In the statement before the 3rd question	“Some people with onychomycosis (nail fungal condition) report being bothered by the following situations. Please answer the following according to how closely you feel these situations describe you.”	“Algumas pessoas com onicomicose (doença fúngica das unhas) referem sentir-se incomodadas pelas situações seguintes. Por favor tenha em conta na sua resposta o quão aproximadamente considera que estas situações o/a descrevem.”
In the statement before the 4rd question	“The following questions ask you to assess your satisfaction with your nail treatment program (Circle one number on each line).”	“As seguintes questões solicitam-lhe que avalie o seu grau de satisfação com o seu plano de tratamento das unhas (Assinale com um círculo um número em cada linha).”

**Table 4:** The translated version and back-translated version of the OnyCOE-t^TM^.

Translated Version (Portuguese EU)	Back-Translated Version (English USA)
*Constrangido e envergonhado*	*“embarrassed, constrained, ashamed”* *These two terms have very similar meanings, presenting a challenge as to discerning which specific, equivalent emotion the researchers wanted to ask respondents about. I chose to translate constrangido as “embarrassed” and envergonhado as “ashamed”. They are similar feelings, but the latter is more serious and implies stronger social judgement and personal culpability.*
*Assinalar*	*Without seeing the visual context of this term, I had translated it as “mark” or “note”, but after seeing that in one instance it was referring to a check box, it became “check the box”, and in other instances, “circle”.*
*Doença fúngica das unhas*	*The literal translation of this term would be “fungal nail disease”, but a quick search revealed that the more common term in English is “fungal nail infection”.*

**Table 5:** Expert committee review of the original and translated version adopted of the OnyCOE-t^TM^.

	Original Version (English USA)	Translated Version Adopted Portuguese (EU)
Question 1	*“1. Please answer the following questions regarding problems you may have had in the PAST 4 WEEKS with your nail fungal condition. Answer each question completely by indicating BOTH: I–HOW MUCH OF THE TIME you experienced the problem, and then II–HOW BOTHERED you were by the problem. (If you are unsure about how to answer a question, give the best answer you can.) TOENAILS”*	*“1. Por favor, responda às seguintes perguntas sobre os problemas que teve nas ÚLTIMAS 4 SEMANAS com a sua doença fúngica das unhas. Responda a cada questão na totalidade indicando: I–COM QUE FREQUÊNCIA sentiu os problemas indicados na tabela, e, em seguida, II–QUÃO INCOMODADO se sentiu com os problemas indicados na tabela. Note que, quando nos referimos a incomodado queremos dizer quão preocupado ou aborrecido se sentiu. (Se não tiver a certeza relativamente a alguma das questões, assinale a resposta que lhe parece mais apropriada.) * *PROBLEMAS COM AS UNHAS DOS PÉS”*
Item (f)	*“Deformity or disfigurement of your toenails”*	*“Deformação ou alteração do formato das unhas dos pés”*
Question 2	*“During the PAST 4 WEEKS, how much of a problem were the following because of your nail fungal condition?”*	*“Considerando a sua doença fúngica das unhas, durante AS ÚLTIMAS 4 SEMANAS, indique quão problemático foram as seguintes situações: (Assinale com um círculo um número em cada linha)”*
NOTE: As the statement is no longer in interrogative form, the items (a) to (p) are now affirmative.		
Item (c)	*“Being embarrassed by the appearance of your nails?”*	*Sentir vergonha com o aspeto das suas unhas.”*
item (d)	*“Feeling self-conscious about the appearance of your nails?”*	*“Reconhecer ou tomar consciência do aspeto das suas unhas.”*
item (f)	*“Feeling that people may see you as unclean or untidy?”*	*“Sentir que as pessoas o/a pudessem ver como pouco limpo/a e desleixado/a.”*
item (o)	*“Concern about concealing your nails, or keeping your shoes on?”*	*“Preocupação em esconder as suas unhas, ou manter-se calçado.”*
item (p)	*“Overall, how much of a problem is your nail condition in your life?”*	*“A sua doença das unhas, em geral, constitui um problema na sua vida.”*
Question 3 Item (f)	*“I feel self-conscious and embarrassed in public.”*	*“Sinto conscientemente o meu problema e sinto-me envergonhado em público.”*

**Table 6:** After the pretest version.

Questions	Problems Identified in Pretest Version
Questão 1 *I. Nas últimas 4 semanas, com que frequência teve os seguintes problemas?* *Item (g) Outros, especifique*	In this question, 4/49 (8%) of the participants attributed the score, but did not specify the problem.
Questão 4 *As seguintes questões solicitam-lhe que avalie o seu grau de satisfação com o seu plano de tratamento das unhas (Assinale com um círculo um número em cada linha)* *Assinale aqui se ainda não teve tratamento:*	Some participants had difficulty understanding whether the treatment plan they had already carried out was framed (for example, systemic treatment with oral medication; topical treatment with varnish or solution; debridement; consultation in dermatology, podiatry). Some participants had already undergone several unsuccessful treatments 13/49 (27%), others had not yet undergone any treatment 24/49 (49%), and as they knew we were doing research in this area, they came in search of treatment for onychomycosis. Most of the 25/49 participants (51%) who reported not having had treatment did not reach the level of satisfaction with the treatment plan; however, approximately 11/49 participants (22%), although they reported not having had treatment, decided to answer questions about the degree of satisfaction.
Overall questionnaire	In all questionnaires, the missing responses were only 1 to 3/49 (2 to 6%). In 6% of the questionnaires, one item was not answered. In 4% of the questionnaires, two items were not answered. In 2% of the questionnaires, three items were not answered.

## Data Availability

The data presented in this study are available on request from the corresponding author. The data are not publicly available due to institutional restrictions.
